# Draft Genome Sequence of the Vanadium-Leaching Bacterium *Pseudomonas chlororaphis* Strain L19

**DOI:** 10.1128/genomeA.00966-17

**Published:** 2018-01-04

**Authors:** Qingjing Peng, Langbo Yi, Lulu Zhou, Qingzhong Peng

**Affiliations:** a Hunan Provincial Engineering Laboratory of Control and Remediation of Heavy Metal Pollution from Mn-Zn Mining, Jishou University, Hunan, Jishou, China; b College of Biology and Environmental Sciences, Jishou University, Hunan, Jishou, China; c Key Laboratory of Mineral Cleaner Production and Exploit of Green Functional Materials in Hunan Province, Jishou University, Hunan, Jishou, China; d The Collaborative Innovation Center of Manganese-Zinc-Vanadium Industrial Technology (the 2011 Plan of Hunan Province), Jishou University, Jishou, China

## Abstract

*Pseudomonas chlororaphis* strain L19, isolated from stone coal soil, has the ability to perform bioleaching to release vanadium ions from mineral ore. Here, we report the draft genome sequence and annotation of the vanadium-leaching bacterium *Pseudomonas chlororaphis* L19. These data provide information for understanding the genomic properties and mineral bioleaching mechanisms of strain L19.

## GENOME ANNOUNCEMENT

Vanadium (V) has been widely used in ferrous and nonferrous alloys to improve their properties of strength, toughness, and abrasion resistance (1, 2). Vanadium compounds exist in over 50 different mineral ores and in association with fossil fuels, particularly stone coal, which is an important source of V (3, 4). The main V extraction processes from stone coal are pyrometallurgy and hydrometallurgy at present, but there are many drawbacks, such as high cost, high pollution, lengthy time investment, and low recovery rate. Many studies have shown that the application of microbial extraction of zinc, copper, and lead from mineral ores is very effective and environmentally friendly (5–7), so bioleaching technology of extracting vanadium from stone coal is being explored.

Our previous use of a microbial community enriched by vanadate from stone coal soil led to successful isolation of a vanadium-leaching bacterium, *Pseudomonas chlororaphis* strain L19. Strain L19 has been deposited in the China Center for Type Culture Collection (CCTCC) under the accession number CCTCC M2015283.

To gain insight into the bacterial leaching mechanisms for vanadium ores, the draft genome sequence of the vanadium-leaching bacterium *Pseudomonas chlororaphis* L19 was determined. Whole-genome sequencing of strain L19 was performed using a MiSeq (Illumina) system by generating paired-end libraries with an insert size of 333 bp and mate-paired libraries with an insert size of 7,539 bp. After low-quality data were filtered, 2,794 Mb and 62 Mb of data were obtained, respectively. The paired-end reads were *de novo* assembled using Newbler version 2.8 (Roche), and the gaps were filled using GapCloser with read mapping information. The coding sequences (CDSs) were predicted by using Glimmer version 3.0 (8). Homologous comparison of all of the genes was performed by BLAST with the NCBI nonredundant public database, KEGG, COG (Clusters of Orthologous Groups), Swiss-Prot, TrEMBL, and GO for function annotation. The tRNAs and rRNAs were identified using tRNAscan-SE and RNAmmer 1.2, respectively (9, 10). Other noncoding RNAs were analyzed by using the Infernal software and the Rfam database (11, 12).

We obtained 5 scaffolds consisting of 16 contigs with a total length of 6,903,284 bp, and the GC content was determined to be 62.79%. It was found that the genome contained 6,305 genes with an average length of 953 bp, and the total length of genes was 6,007,953 bp, which makes up 87.03% of the genome. Among the whole-gene sets, 5,473 CDSs were involved in the 23 COG groups and 945 CDSs were involved in the 12 metabolic pathway KEGG groups. In addition, 59 tRNAs with a total length of 4,514 bp, 2 rRNA operons, and 86 other noncoding RNAs (ncRNAs) were also determined in the genome. A detailed comparison of the L19 genome with the genome of other *Pseudomonas chlororaphis* strains has shown that L19 has 8 specific genes, which may provide some insights into the molecular mechanism about bioleaching of vanadium.

### Accession number(s).

The draft genome sequence of *Pseudomonas chlororaphis* strain L19 has been deposited at DDBJ/EMBL/GenBank under the accession number LNTS00000000. The version described in this paper is the first version, LNTS01000000.
